# A Sustainable Model for Healthcare Systems: The Innovative Approach of ESG and Digital Transformation

**DOI:** 10.3390/healthcare12020156

**Published:** 2024-01-09

**Authors:** Anastasios Sepetis, Fotios Rizos, George Pierrakos, Haralampos Karanikas, Daniel Schallmo

**Affiliations:** 1 Postgraduate Health and Social Care Management Program, Department of Business Administration, University of West Attica, 12244 Athens, Greece; gpierrakos@uniwa.gr; 2 Department of Business Administration, University of West Attica, 12241 Athens, Greece; frizos@uniwa.gr; 3 Department of Computer Science and Biomedical Informatics, University of Thessaly, 35131 Lamia, Greece; karanikas@uth.gr; 4 Institute for Entrepreneurship, University of Applied Sciences Neu-Ulm, 89231 Neu-Ulm, Germany; daniel.schallmo@hnu.de

**Keywords:** ESG factors, digital transformation, sustainable finance, healthcare system, health, open innovation

## Abstract

In recent years, the globe has faced a series of topics of growing concern, such as the COVID-19 pandemic, the international financial crisis, rising socio-economic inequalities, the negative outcomes of greenhouse gas emissions, which resulted in climate change, and many others. Organizations worldwide have confronted these new challenges of sustainable finance by incorporating environmental, social, and corporate governance (ESG) factors and digital transformation (DT) in their innovation business strategies. The healthcare sector represents a large share of the global economy (about 10% of global economic output), employs a large number of workers, and needs to rely more on an open innovation model where interested parties, especially patients, are going to have a say in their own well-being. Thus, it is imperative that healthcare providers be efficient, effective, resilient, and sustainable in the face of significant challenges and risks. At the same time, they must offer sustainable development goals and digital transformation to healthcare users through limited governmental resources. This study investigates the role, importance, and correlation of ESG factors and digital transformation to the sustainable finance of healthcare systems through an innovative model. The main purpose of the paper is to present the already implemented ESG and DT factors in the healthcare sector and to propose a mutual and combined implementation strategy based on common evaluation tools, methods, and actions. A set of proposed actions and strategies are presented for the sustainability and resilience of the healthcare sector.

## 1. Introduction

Nowadays, the globe is facing numerous challenges to a degree that has never been seen before. For example, we are facing serious environmental issues (such as the constant climate change as a result of greenhouse gas emissions and water and air pollution), public health issues (such as various severe pandemics, with COVID-19 being the most recent), financial crises, and social issues (such as the increase in inequalities and the spread of poverty in many countries [1].

These challenges have also affected the investment practice and their way of operation and have led organizations to adopt and implement ESG models that can strengthen business sustainability and performance [2] but also play a part in the contribution to the development of new sustainable finance models that are capable of meeting the market needs, demands, and expectations from the stakeholders and shareholders [3,4,5,6,7,8,9,10,11]. In addition, the adoption of an ESG policy has been shown to affect business performance regarding sustainable business investments [12].

One of the sectors that is directly affected by these changes and needs to implement ESG factors in its operations through innovative digitalization strategic models is the healthcare sector [13,14], which represents a large share of the global economy, estimated at about 10% of the global economic output [15].

The healthcare sector must respond to these new sustainable business challenges by integrating ESG factors into their sustainable business challenges. The general philosophy of ESG is in a straight line with the concept of sustainable healthcare without harm and infers a responsible approach towards the environment (e.g., specific actions for the decrease in greenhouse gas emissions and water consumption, use of renewable energy sources, etc.), compliance with social responsibility (e.g., policies to fight discrimination incidents in the workplace with regards to human rights, occupational hygiene and safety measures and protocols, etc.), and proper implementation of corporate governance (e.g., establishment of anti-bribery policies and procedures, handling whistle-blower schemes, etc.) [16,17]. Moreover, it employs a large number of workers and plays a significant role in decreasing the damage and degradation of the natural environment due to its continuous 24/7 operation and constant energy consumption [15,18].

Another approach that organizations and stakeholders use in order to overcome the negative consequences of the pre-mentioned challenges is through digital transformation. Organizations are integrating digital transformation models parallel to sustainable business models in order to incorporate new knowledge in several business sectors that arise from external parties, such as academic institutions, policy makers, NGOs, etc. [19,20]. Unsurprisingly, in the sustainable development era, the majority of companies have already found themselves in the middle of significant digital transformation initiatives ranging from large-scale, cross-organizational digital initiatives to smaller digital projects. Digital transformation, including the contexts of strategy-oriented digitalization and digitization, influences all sectors of society, especially economies, and additionally, different sectors have connected their business successes to the application of cognitive technologies [21]. Moreover, researchers have revealed several popular ways of promoting digital transformation in business practice [22]. In general, digital transformation has fundamentally changed organizational processes, core products and services, and existing organizational strategies. In addition, the pace of digital innovations’ introduction into business processes affects directly the level of their competitiveness and the respective investment attractiveness, creates innovative opportunities for sustainable business challenges, and affects notably the firms’ value [23,24,25]. For that reason, digital transformation is one of the key factors in bringing sustainable development goals into the new conditions, urges innovative and sustainable models in all areas of socio-economic activity, is aligned with the main principles of the new Green Deal, and can be used in order to achieve UN Sustainable Development Goals (SDG) [26].

According to various studies, we have found that the concept and implementation of ESG and DT factors are considered and managed as separate entities, even though they both contribute to sustainable development [4,6,7,8,14,27,28,29,30]. Thus, we attempt a first approach on this matter in order to investigate a potential correlation between these concepts and the implementation of a proper strategy [31]. What drew our attention and motivated us to develop this paper was the fact that there is no actual reference on how ESG and DT factors and initiatives are applied in the healthcare sector as a mutual and combined implementation strategy with regards to their specificities. So, the main research question that follows our paper is:

**RQ “How can ESG and DT factors apply in the healthcare sector as a mutual and combined implementation strategy, using common evaluation tools and methods?”**

Thus, we performed a narrative literature review in order to investigate the ESG and DT factors that are applicable in the healthcare sector and how they could be implemented as a new innovative business strategy. The main research objectives that this paper is trying to answer, but are not limited to, are:

- What is the current theoretical background that arises from the literature review regarding the implemented ESG and DT factors in the healthcare sector?
- What are the ESG and DT factors that have an impact on healthcare sustainability?
- Are there any methodologies used for mutual measurement, evaluation, and reporting of ESG and DT factors in the healthcare sector?

In addition, this paper is deemed to be one of the studies to highlight the importance of ESG and DT synergies that occur and provide vital benefits in the healthcare sector in terms of sustainability. Based on the above statements, this research is structured as follows: In Section 2, the theoretical background and a brief presentation of the methodology of this paper are presented. In Section 3, the ESG and DT factors that are currently implemented in the healthcare sector are presented, along with the success factors and proper steps for an effective digital transformation implementation. Section 4 proposes a specific methodology for a mutual ESG and DT model in the healthcare sector. Finally, Section 5 presents the concluding comments and remarks, along with the research gaps, the potential need for future academic research development, and the implications of this paper.

## 2. Theoretical Background

### 2.1. Methodology of Our Research

In order to perform comprehensive research, we chose to use the systematic literature review’s guidelines, and we followed specific steps for the literature review process in accordance with Yu and Watson [32] and Tranfield and Smart [33]. After the definition of the research question and the respective objectives, we developed and validated a review protocol that included the inclusion and exclusion criteria and the methods that were going to be used in the review process. A specialized work group was formed, consisting of two review authors—experts in the field of digital transformation, one review author—experts in the field of ESG and sustainability in the healthcare sector, and one coordinator. The review authors performed a parallel independent assessment of the manuscripts, and in case of a disagreement or dispute, the coordinator was responsible for giving a solution.

For our search, we used the online databases of Google Scholar, CORE, and PubMed, which contain journal articles as well as “gray literature,” such as conference proceedings and reports, and reviewed the first 15 pages of search results from the last two decades. The search was performed using the following terms, keywords, and abbreviations: ESG, ESG in healthcare, digital transformation, digital transformation in healthcare, digital health, sustainability in healthcare, sustainable finance in healthcare, innovation in healthcare, and only publications written in English.

First of all, we identified and selected the initial group of 357 studies that were screened through their titles, the context of the abstracts (for papers), and the table of contents (for reports and non-academic publications) in order to investigate the relativity of the papers based on the research question and relative objectives. The next step was to select the papers, publications, and reports that were close enough to answer them. A total of 169 studies were deemed relevant, and we obtained the full-text article for quality assessment. Then, we skimmed through the full-text articles to further evaluate the quality and eligibility of the studies. After careful review, a total of 75 studies were excluded, and altogether, we included a total of 94 studies in this research that meet our inclusion and exclusion criteria. After analyzing and synthesizing the existing information from the selected studies, we codified it into specific concepts such as ESG in healthcare, DT and digital transformation in healthcare and sustainable finance, and then presented our findings. Afterwards, we concluded with the suggestion of a new ESG and DT model with a mutually combined implementation strategy in the healthcare sector. The implications of this research are quite significant for all healthcare actors (e.g., patients, healthcare providers, health institutions, academia, healthcare experts and executives, governments, etc.) because it presents the ESG and DT factors that are currently being used, their synergies, and how they can be evaluated as one entity in order to fulfill the expectations of the stakeholders and try to identify the problems that affect the healthcare sector.

### 2.2. ESG and DT Factors in Green Deal and Digitalizatin Era

The concept of sustainable finance and the ESG and DT factors are directly related to the concept, general philosophy, and policy of Sustainable Development Goals (SDGs) [28]. More specifically, in recent years, the shift towards the Green Deal and digitalization era has accelerated and spread rapidly and continuously worldwide as a new expectation for organizations. Thus, the financial market, ESG, and DT factors are linked with the latest business models and strategies in many ways, such as the level of competitiveness, the proper profit–cost policies, and the organizations’ shareholder value.

Today, governments, the public, and organizations are more interested in sustainability issues such as the climate crisis, global warming, reduction in global emissions, resource depletion, air, water, and land pollution, quality of life, income disparities along with social inequalities, the growing of stakeholder activism, and the lack of mutual and universally accepted legislation, which have resulted in the imperative need for sustainability implementation, from an optional state to a must-have concept in the core business [3,4,20,27,34,35]. This also applies to the private sector, where shareholders, stakeholders, investors, and even consumers demand responsibly derived products and services with the minimum environmental footprint, its role in answering these challenges, and how society should measure business success apart from the financial aspect. On top of that, sustainable businesses are rewarded by customers for their loyalty, investors for their capital, and employees for their total engagement. Thus, more investments are reflecting the impact of ESG risks on risk-adjusted returns, and their continuous effort to align with the values of sustainable finance is crucial for the public and private sectors, even at a governmental level [36]. Furthermore, investment funds that meet ESG and DT criteria are found to be more stable compared with other types of collective investment undertakings, and investors are less likely to withdraw their investments after a negative performance [27,29,37,38,39]. In addition, sustainable finance taxonomies (such as Sustainable Finance Taxonomy–Regulation [EU] 2020/852 in Europe) are vital for sustainable finance and support the achievement of high-level goals such as the UN SDGs. However, studies mention some limitations that arise from the classification and comparison of these taxonomies, including the failure to use appropriate and measurable sustainability performance indicators and a lack of verification of accomplished sustainability benefits [40].

On the other side, studies have shown that digital technologies create new innovations and affect their openness degree [25], with a direct impact on entrepreneurial initiatives, new assets, and the creation of new ventures [41,42], gain economic and social advantages, and also promote economic and social well-being [43]. In addition, one of the factors that is proven to have a positive relationship with economic growth, organizational development, competitiveness, and sustainability is innovation [44]. At the same time, a holistic approach to digitalization has become vital for all kinds of institutions and organizations, and the economic impact of DT, the future policy directions, and the ramifications derived from DT (including the contexts of strategy-oriented digitalization and digitization) affect all sectors of society and the improvement of quality of life [45,46]. According to Caputo et al., 2021, the terms ‘digital transformation’ and ‘strategy-oriented digitalization’ contain several overlapping characteristics, and they argue that certain digitalization-level initiatives (e.g., the digital process model) may also demonstrate strategy-oriented perspectives [47]. Digitalization initiatives, especially in digital maturity, strategy, transformation, implementation, and completion, offer a way for companies’ optimization of sustainable financial evaluation. Thus, the implementation of digital technologies and their ability to affect ESG goals are becoming more convergent because improved data collection, reporting, and analysis will benefit every part of the business [34].

A market section that must respond to these new challenges by integrating ESG and DT factors into their business strategies and financial statements is the healthcare sector.

### 2.3. Role of ESG and DT in the Healthcare Sector

It is commonly known that the healthcare sector is undergoing profound changes due to the increased technological innovations combined with automation and miniaturization, which have led to the creation of large amounts of data production, and this exponential increase should be managed accordingly [13,48,49,50]. The potential of “big data” for improving healthcare systems is immense, and simultaneously, a wide range of urgent challenges for the EU sustainable development policy evaluation have arisen [51].

Furthermore, ESG and DT factors’ motives affect the healthcare sector, where their basic goals are to provide good health, responsiveness to the expectations of the population, and fairness of financial contribution under sustainable finance criteria [52]. The healthcare sector is constantly trying to achieve better financial results, provide quality services with synergies, and adapt to the new offer conditions of ESG and DT factors [53]. Additionally, these factors try to create a center of attention for consumers, investors, and private institutional sustainable financial stakeholders and private sustainable investors, and as a result, healthcare systems associate unescapably at an increasing rate with sustainable finance strategies and sustainable financial stakeholders.

The Big 4 (PwC, Deloitte, EY, and KPMG) have emphasized the value, importance, and numerous benefits of applying ESG factors in the health sector [36,54,55,56,57]. In addition, researchers have found that public health facility managers adopt initiatives and ESG factors to save money, improve facility operations, increase employee satisfaction and retention, manage risks and explore business opportunities, comply with the regulatory framework, enhance facility operations, achieve performance excellence, and present or report environmental and corporate social responsibility and governance issues [16,17]. Moreover, researchers state that the correct implementation of ESG and DT initiatives and measures leads to the improvement of service quality and increases the business value of healthcare providers, as well as the indicators that are proven to have a positive impact on their operational management, investments, and financial management. These include increased profit margins; long-term licenses; promotion of goodwill, brand, and reputation; strengthening of capital investments for the development of new innovations (capital innovation); strengthening capital investment in know-how and development of human skills (human intellectual); ensuring stable, smooth, and low interest rates (good risk profile); reducing financial risk; and so on [48,58,59,60,61].

It is interesting to point out that European healthcare systems and databases are diverse and fragmented, and there is a lack of sustainable finance taxonomy for the harmonization of data formats, processing, analysis, and data transfer, which leads to inconsistencies and lost opportunities. Sustainable finance legal frameworks for data sharing are changing, and both ESG and DT criteria need synergies. Financial and insurance analysts, investors, researchers, and society in general need improved methods, tools, and training to generate, analyze, and query data effectively to evaluate ESG and DT factors. Thus, it is imperative to be efficient, effective, resilient, and sustainable in the face of significant challenges and risks and, at the same time, provide sustainable development goals to healthcare users through limited governmental resources. In addition, due to economic crises, where the reduction in funding for healthcare requires optimal utilization of environmental costs (e.g., energy consumption, waste medical), the social benefits of patients should be refocused according to ESG and DT criteria. Thus, more has to be conducted in order to achieve sustainability and resilience in the healthcare systems and the healthcare sector in the post-COVID era, such as proper organizational changes, suitable health policy reformation, and the implementation of operational procedures and management models [62]. To this end, in this paper, we suggest corrective and structural changes that need to be performed through an open innovation process with mutual and common integration of ESG and DT criteria in the healthcare system [63,64].

In the next section, we will present the findings of our research and, more specifically, the currently implemented ESG and DT factors that concern the healthcare sector and affect sustainable finance, the actions required for a successful digital transformation, and the steps to be taken for the implementation of a mutual ESG and DT strategy.

## 3. Findings and Analysis

### 3.1. ESG Factors in the Healthcare Sector

According to a study [65], a general benefit for organizations that have implemented ESG criteria is to have high ratings, as well as to be more competitive and demonstrate abnormal returns, which often leads to higher profitability and dividend payments, specifically when compared to other low ESG organizations. In addition, high-ESG-rated organizations experienced fewer peculiar risk incidents, such as an important drawdown. On the other hand, organizations with low ESG ratings were more likely to experience these incidents. Finally, high-ESG organizations had fewer volatile earnings and less systematic volatility, lower betas, and lower costs of capital than low-ESG-rated organizations. In general, an organization’s compliance with ESG principles may lead to better:

- Understanding about various concerns towards the environment, such as reduction in the harmful impact (E-principle).
- Social responsibility, such as responsible investments in local communities, local providers and suppliers, and better working conditions (S-principle).
- Corporate governance actions, such as the implementation of anti-corruption policies and measures and improving corporate culture (G-principle).

Healthcare facilities have integrated and implemented ESG activities into their operations and are trying to determine an eco-friendly profile for stakeholders, mostly in society, through the use of sustainability reports and scorecards. From our research, we have collected and presented the most commonly mentioned ESG factors that healthcare providers are measuring and demonstrating.

#### 3.1.1. Environmental: Conservation of the Natural World

The healthcare sector plays an important role in the preservation and restoration of the natural environment due to its 24/7 operation, constant energy consumption, and enormous production of healthcare and medical waste [18,35]. Although the healthcare sector is thought to be one of the greenest industries, its global carbon footprint is measured to be 4.4 percent of the world’s total [66]. Thus, this sector is crucial for the implementation of environmental factors that will be associated with the quality and functioning of the natural environment. According to our research, the proposed environmental factors to consider are:

- climate change and carbon emissions;
- intention to lower greenhouse gas emissions and CO_2_ footprints;
- air and water pollution;
- energy consumption and efficiency;
- water scarcity;
- biodiversity;
- use of clean technology;
- deforestation (existence of responsible practices across the value chain);
- resource depletion;
- use of renewable energy sources that contribute less to climate change;
- waste management (adoption of circular economy principles, implementation of quality management systems, and ISO certifications);
- limits on harmful pollutants and chemicals;
- disclosure of information on all environmental policies; and
- publication of a carbon or sustainability report.

The expected positive benefits are outcomes such as the reduction of costs and reputational risks and the improvement of profitability in terms of better energy efficiency so as to achieve sustainable finance principles.

#### 3.1.2. Social: Consideration of People and Relationships

Healthcare systems, which are an essential portion of social protection systems, must attain and preserve the social health and well-being of communities and society in general. Our research proposes the following social factors for consideration:

- Patients’ satisfaction;
- data protection and privacy of all interested parties and stakeholders;
- anti-discrimination policies;
- employee engagement (training and development of staff and increasing their involvement);
- community environmental impacts;
- community relations (good relations with local communities and other organizations related to the healthcare sector, such as the Patient’s Association);
- healthcare employee relations (improvement of their working conditions);
- human rights;
- labor relations and standards (no questionable workplace safety or child labor);
- operation of ethical supply chains;
- policies to protect against sexual misconduct;
- fair (living) wages (labor standards that ensure fair wages and the protection of human rights);
- health and safety measures and standards (safe and healthy working conditions for healthcare employees);
- conflict management;
- data hygiene and security;
- mental health;
- ethical product sourcing.

The expected positive benefits are increased healthcare organizations’ productivity, decreased turnover, a boost in the employee’s morale, and better management of reputational risks. Following these recommendations also makes it easier to work without social pressure from stakeholders, improving the patient’s experience and satisfaction.

#### 3.1.3. Corporate Governance in Healthcare

In general, governance factors in the healthcare sector emphasize policies and how healthcare providers are governed by clarifying the responsibilities, rights, and expectations of stakeholders so that interests are met and a consensus is achieved on a long-term strategy. Furthermore, governance factors cover aspects such as executive leadership, board independence, shareholder rights, corruption, and bribery, and the way in which healthcare providers include environmental and social factors in their policies and procedures, such as management structure, management remuneration, transparency, business integrity, lobbying, rights of and relations with shareholders and relations, long-term strategy, internal control and audit, etc. Our research proposes the following governance factors for consideration:

- Board composition (embraces diversity on the board of directors);
- audit committee structure;
- executive compensation guidelines;
- political contributions (in the case of the private sector);
- whistle-blower schemes;
- embrace corporate transparency;
- hiring and onboarding best practices;
- lobbying;
- tax strategy;
- risk management;
- protecting shareholder interests with special attention to patients;
- health and business ethics;
- prevention and management of bribery and corruption incidents via specific guidelines, e.g., anti-corruption policies;
- corporate culture and code of conduct.

The expected positive benefits from the implementation of the above-mentioned measures and policies are the consideration of all shareholders’ interests, the improvement of the overall management, the reduction of any financial surprises, and the achievement of better social acceptance as a result of wealth being fairly distributed.

It has become clear that by implementing the above-mentioned ESG factors, healthcare providers can contribute to a great extent to the conservation of the environment, the preservation of social health and of society in general, the improvement of corporate governance in healthcare facilities, and, on a larger scale, to achieving the SDGs. Another set of factors that contribute to the sustainability of the healthcare sector are DT factors.

### 3.2. Digital Transformation Factors in Healthcare

The introduction of DT in the healthcare sector has gained significant interest and has been a field of constant application and development for the last 20 years [50,67]. In today’s business environment, where synergies are vital, organizations need to exchange knowledge, ideas, and even employees and experts to overcome organizational boundaries so as to develop new sustainable business models. The meaning of digital transformation, except for the adaptation of digital technologies to improve processes and services, also includes new business models such as the open innovation process, which played a moderating role during the COVID-19 pandemic due to the sharing of scientific knowledge, findings, and results of medical research, especially for the development of vaccinations [20]. More specifically, despite the fact that healthcare innovation literature is rather limited to the management of the COVID-19 pandemic, studies state that there is a connectivity between innovation in healthcare and patients’ satisfaction, profitability, and enhanced research and development performance [20,68,69].

It is worth mentioning that an organization’s performance is affected by new technologies and the connectivity of all stakeholders across the value-added chain. The healthcare sector has adopted DT technologies to provide secure and high-quality services and improve their operational efficiency by enabling clinical and administrative activities associated with the assessment, transmission, evaluation, and precision of medical treatment [30,70]. The main healthcare technology-related areas of interest to researchers are:

- Integrated management of information technology in healthcare [50];
- Health information technology [71,72];
- medical images [49,50];
- electronic medical records [50];
- electronic health records [73];
- access to e-health [50];
- telemedicine [50];
- privacy of medical data [49];
- mobile technologies, applications, wearables, and software platforms [71,72,74];
- advances in health platforms and data analysis [75,76,77,78];
- big healthcare data [79];
- artificial intelligence [80];
- online health communities [81,82].
- cognitive technologies [21]

The most interesting approach to digital sustainability from healthcare providers is the application of digital transformation factors and activities. However, very few publications can be found in the literature that address the steps to be followed in order to achieve successful digital transformation implementation in the healthcare sector.

### 3.3. Successful Digital Transformation Implementation in Healthcare

Schallmo and Williams [22] state that there are multiple ways to promote DT. These include digital strategy, digital business models, digital products and services, digital processes, and digital maturity models (Table 1). According to Morakanyane et al. [83], there are seven factors and 23 subfactors needed for an organization to achieve successful DT, as shown in Table 2. These must be applicable in the healthcare sector.

Moreover, Osmundsen et al. [84] performed a systematic literature review of digitalization that included 69 papers, 26 from journals, and 43 from conferences. Drivers and objectives are the attributes and goals that initiate and influence DT factors. Success factors are essential organizational elements for accomplishing digital transformation criteria, and implications are the effects organizations experience as a result of digital transformation.

As mentioned before, several publications have appeared in recent years documenting ESG and DT factors and approaches as individual entities and concepts, although they have many similarities and common characteristics and evaluation methods. According to our research, it was observed that there is no methodology for joint ESG and DT evaluation in the healthcare sector. Thus, in the next section of this paper, a proposed detailed strategy for holistic and mutual ESG and DT evaluation is presented.

## 4. Proposed Methodology for a New Mutual ESG and DT Model in Healthcare Sector

### 4.1. ESG and DT as Synergy Concepts

The impact of digital technology is visible in many aspects, such as the reduction in operational costs and human errors. Notably, manually performed ESG-related activities face certain problems, such as uncertainty in data accuracy and limited visibility, reporting, data saving, and benchmarking. This is relevant for digitization, which allows organizations to transform their manual workflow and processes in a more efficient, accurate, and consistent way.

Studies have demonstrated the close and important connection between ESG and DT as both activities seek to create a new modern (sustainable) business and operational model and framework and fulfill the expectations of interested parties and stakeholders along with the outcomes for society and the environment [28,29]. Indicatively, we can see their connection in the improvement of data collection, reporting, and analysis and in achieving certain priorities and objectives such as data protection and workforce productivity regulatory compliance [29,34]. Moreover, ESG and DT correlation helps identify cost-saving options in particular segments of a process or service that may be considered wasteful, and additionally, the transformation, implementation, and compliance of ESG factors are quicker and more effective with digital technologies [85].

Thus, it is critical to have a mutual concept of ESG and DT in terms of implementation, evaluation, measurement, and reporting. Our methodological model consists of the following steps:

- Development of a mutual ESG and DT strategy with respect to open innovation;
- implementation of a mutual ESG and DT strategy;
- determine the ESG and DT framework, standards, and reporting to be applied;
- select the proper tools for mutual evaluation of f ESG and DT actors such as the proposed KPIs that are mentioned in Section 4.5.

### 4.2. Indicative Steps to Be Followed for the Implementation of Mutual ESG and DT in the Concept of an Innovation Strategy

It is very important that all actors and stakeholders are involved in the adoption of innovations and new ideas. The proposed success factors and challenges for innovation initiatives, based on the ESG and DT approaches in healthcare systems, are:

- Create new ideas from an interdisciplinary work group: The participation of professionals with different professional, academic, and cultural backgrounds is essential for the development of new ideas and initiatives in the healthcare sector, with good knowledge, expertise, and experience in ESG and DT factors.
- A clear definition of the goals and objectives for innovation and initiatives (including the vision, strategic culture, and principles) towards sustainability.
- Identification and definition of certain barriers to innovation such as the training of healthcare professionals so as to have more skills and qualifications and their participation in networks/clusters, the regulation restrictions that arise from national, European, and international legal frameworks, with regards to ESG and DT factors.
- Innovation initiatives should be specific, measurable, achievable, relevant, time-bound, evaluated, and reevaluated/readjusted.
- Support from distinguished experts, healthcare executives, esteemed high-profile leaders, and other specialized executives could drive all interested parties to the completion of the ESG and DT innovation initiatives.

### 4.3. Implementation of a Mutual ESG and DT Strategy

According to our research and experience, we have identified and proposed the following steps to be performed by healthcare providers for a smooth and proper implementation of ESG and DT factors:

- Create a work position in the organization along with the respective job description and work instructions.
- Appoint a qualified person for the ESG and DT strategies.
- Perform an identification, assessment, and evaluation of ESG and DT risks associated with investments in the healthcare sector.
- Acquire data so as to provide insights on risks related to climate change.
- Cooperate with external professionals and experts for additional support.
- Provide an overview of ESG and DT best practices, trends, relative norms, legislation, and regulations in the respective sector of operation.
- Develop a due diligence report based on ESG and DT factors and the current legislation to present information with regards to, investment decision making, negotiations and integration to all stakeholders.
- Develop best practices, golden rules, guidelines, and checklists covering every related aspect.
- Develop proper internal procedures, processes, and policies, along with planning activities for ESG and DT due diligence.
- Perform the proper risk assessment process in order to identify, analyze, evaluate, and manage operational ESG and DT risks and opportunities.
- Plan and perform specialized internal training for all involved personnel.

### 4.4. Determine the ESG-DT Sustainability Framework, Standards and Reporting

Organizations such as hospitals need to demonstrate their compliance with ESG, including DT factors, based on a specific framework and standards, as well as identifiable guidelines, principles, criteria, requirements, data collection methodology, and reporting to be available to all stakeholders in a reliable, transparent, and comprehensive way. For that reason, NGO organizations such as the Global Reporting Initiative (GRI), the Sustainability Accounting Standards Board (SASB), International Integrated Reporting Council (IIRC), and other NGO institutional organizations provide a respectable framework for ESG, and institutional organizations such as the European Financial Reporting Advisory Group (EFRAG) and the International Sustainability Standards Board (ISSB) provide standards for ESG reporting.

As far as the ESG and DT legislation is concerned in healthcare systems (particularly in the private sector), in the last few years, significant progress has been made with regards to regulations and standards regarding ESG criteria, especially in Europe, where the European Commission published the Corporate Sustainability Reporting Directive (CSRD), which has been in force since the beginning of 2023 and provides a general framework and set of rules for the disclosure of information about the risks and opportunities arising from social and environmental issues and the impact of their activities on people, the environment, and other sustainability issues. In addition, there is the European Sustainability Reporting Standard (ESRS) by the European Financial Reporting Advisory Group (EFRAG), which takes into account the EU Taxonomy Climate Act, and on an international level, there are the International Financial Reporting Standards (IFRS) that have established the International Sustainability Standards Board (ISSB). However, research suggests that these new facts need more research on scientific and legislative topics. In our relevant research, we have identified a lack of common reporting for both ESG and DT factors, and we believe that a specialized reporting ranking should be developed that will be incorporated in the healthcare sector so as to be treated and reported mutually, according to the needs and expectations of the interested parties and in accordance with the organization’s characteristics.

### 4.5. Tools for Mutual Evaluation of ESG and DT Factors

There is considerable interest in ESG performance assessment in the business world [86,87]. Some studies state that ESG and DT evaluation with regards to the healthcare sector is inadequate and lacks a common base on what to measure, who to involve, and how to evaluate [88]. ESG and DT key performance indicators (KPIs) are a proper way for a healthcare provider to track figures to understand the ESG and DT impact of their operations. ESG and DT KPIs will also provide managers and investors with an image of the risks their investments and funds confront and what measures should be taken. This paper presents the most commonly used KPIs that are used in the healthcare sector in order to measure and evaluate the strategic goals and planned objectives and to get an overview of how healthcare facilities are performing (Table 3).

**Figure 1:**
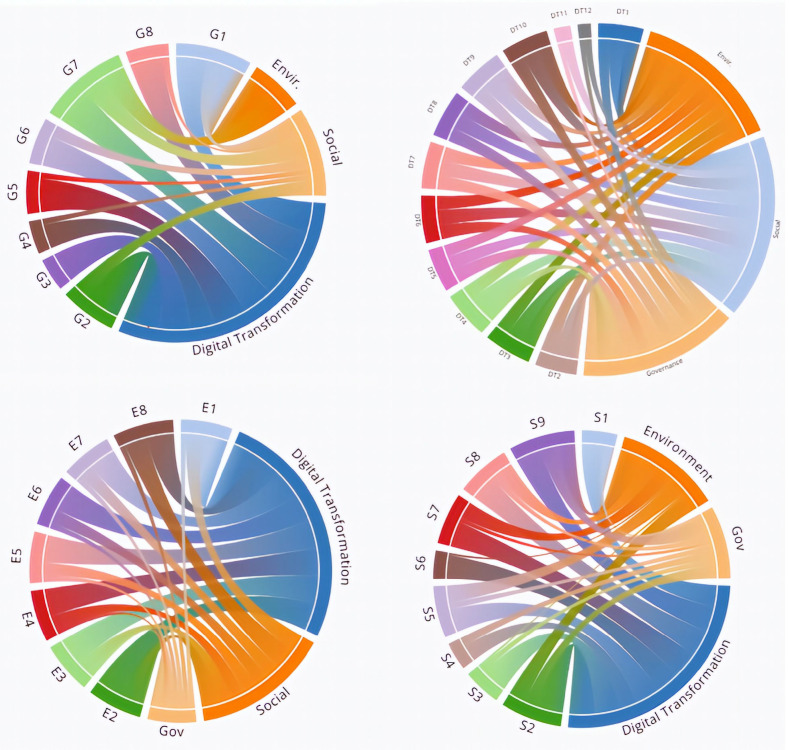
Correlation between ESG and DT KPIs. Source: Derived from our research.

In addition, this paper tries to make a first attempt to show the correlation between the above-mentioned KPIs based on the author’s empirical research. From this graph, it can be seen that most of the KPIs are linked and have an immediate correlation. What is interesting to point out is that DT KPIs seemed to have a correlation with almost all the other KPIs. These findings are not conclusive, and our analysis does not enable us to determine new KPIs that can measure and evaluate, on a mutual basis, ESG and DT factors. Further research needs to be carried out due to the promising findings presented in this paper, and the work on the remaining issues is continuing and will be presented in future papers (Figure 1).

Moreover, an integrated sustainability scorecard should be available that will refer to both ESG and DT to record and monitor information, data, and the implemented initiatives and to perform improved criteria and any corrective or preventive actions.

Sustainable finance legal frameworks with regards to data sharing are growing, and ESG and DT criteria need synergies. Financial analysts, investors, researchers, and citizens need improved methods, tools, and training to generate, analyze, and query data effectively to evaluate ESG and DT factors. An integrated module for the healthcare sector in terms of ESG and DT factors should be developed, pilot tested, and improved to fill this gap [40].

## 5. Conclusions

It has become clear that the healthcare sector faces a set of challenges in ensuring the equivalence, consistency, reliability, resilience, transparency, and quality of the data and relative metrics. ESG and DT factors related to the healthcare sector are proven to have a very important role and affect sustainable finance. Healthcare providers should incorporate meaningful and relevant ESG and DT data into investment decision making and disclose ESG and DT information through clear, practical, and specific guidelines. Also, the proper identification, evaluation, and implementation of ESG and DT activities and initiatives and the use of a common strategy based on mutual ESG and DT evaluation models and reporting methodologies in terms of sustainable finance are essential for the sustainability of the sector and are proven to attract more investors and funds, but they also create opportunities for improvement to attract green loans, investors, and funds.

This study has presented the current theoretical background regarding the implemented ESG and DT factors in the healthcare sector. To our knowledge, this is the first study that attempts to identify, analyze, categorize, and evaluate mutual ESG and DT factors related to sustainable finance that can be applied in the healthcare sector. Based on the findings of our research, it can be concluded that ESG and DT factors are implemented and performed systematically in the healthcare sector, but not to a great extent, and are handled as different entities in terms of measurement, evaluation, and strategic planning in general. Our paper presents an innovative view of a proposed methodology that can be readily used in practice, which combines the two concepts into one strategy with specific steps for the mutual implementation, evaluation, measurement, and reporting of ESG and DT factors. This holistic approach to ESG and DT will ensure that all crucial components of materiality from ESG and DT in healthcare have been taken into account, establish the proper mutual ESG and DT risk assessment models, and identify, evaluate, and implement ESG and DT international best practices. The findings suggest that this approach could also be useful for healthcare providers who want to alter the existing strategic approach of their facility into a sustainable one, and it can provide guidance on the steps to be developed.

Moreover, this paper demonstrates and proposes that exclusive KPIs can be combined and used as an overall tool for the parallel measurement, evaluation, and reporting of ESG and DT factors and initiatives. Clearly, further research is needed to validate the KPIs’ correlation that we presented in this paper.

This paper aims to be a beacon of future research on ESG and DT factors in the healthcare sector in terms of sustainable finance. The limitations of our paper are the outlined principles and proposed research methodology that require further in-depth investigation based on a systematic literature review, and there may also be more factors that influence ESG and DT in the healthcare sector than those investigated and theoretically introduced. Although various factors have been demonstrated per category, more field research needs to be carried out so as to perform an ESG and DT factors’ mapping in both the public and private healthcare sectors in different healthcare systems.

In addition, specialized research is needed for the development of an integrated module for healthcare systems in general in terms of ESG and DT factors and the respective financial model that will evaluate the financial significance of these factors and their correlation with sustainable investments.

## Figures and Tables

**Table 1:** Steps for a successful digital transformation implementation.

**Determine digital trigger**	- Knowledge in the triggers’ type - Knowledge in the inducers’ type
**Determine digital drivers**	- Determination of digital technologies to leverage - Determination of skill and capabilities required - Determination of other resources impacting required - Demonstration of strong digital leadership traits
**Establish digital organization**	- Establishment of digital innovation functional structure - Creation of digital innovation implementation structure
**Determine impacts**	- Definition of expected customer facing impacts - Determination of realized customer facing impacts - Definition of expected organizational facing impacts - Determination of realized organization facing impacts - Determination of measure of impacts
**Determine transformed areas**	- Determination of transformation opportunities - Identification of target transforming areas - Building digital transformation initiatives
**Develop digital vision**	- Perform digital present awareness - Formulation of a digital future - Development of a specific digital strategy - Establishment of a digital communication strategy
**Cultivate digital culture**	- Ensure shared conceptualization of digital transformation - Exhibit strong organizational leadership traits - Adopt good governance practices

Reprinted from Morakanyane, R., O’Reilly, P., McAvoy, J., and Grace, A. (2020) [83]. Determining Digital Transformation Success Factors. Hawaii International Conference on System Sciences.

**Table 2:** Factors for digital transformation.

**Success factors**	- A supportive organizational culture - Well-managed transformation activities - Leverage external and internal knowledge - Engage managers and employees - Grow IS capabilities - Develop dynamic capabilities - Develop a digital business strategy - Align business and IS
**Drivers**	- Customer behaviour and expectations - Digital shifts in the industry - Changing competitive landscape - Regulative changes
**Objectives**	- Ensure digital readiness - Digitally enhance products - Embrace product innovation - Develop new business models - Improve digital channels - Increase customer satisfaction and dialogue
**Implications**	- Reformed IS organization - New business models - Effects on outcome and performance

Reprinted from Osmundsen, K., Iden, J., and Bygstad, B. (2018) [84]. Digital Transformation: Drivers, Success Factors, and Implications. In MCIS. Reprinted with permission.

**Table 3:** ESG and DT KPIs used in the healthcare sector.

**Environmental KPIs**	E1: % of energy in kWh from renewable energy sources as of total energy consumed
	E2: % of energy in kwh from combined heat and power generation as of total energy consumed
	E3: energy efficiency energy consumption total
	E4: GHG emissions GHG emissions total corporate
	E5: energy consumption
	E6: water consumption
	E7: waste consumption
	E8: medical waste as a % of the total produced waste
**Social KPIs**	S1: absence rate (KPI Ex: number of worker-days lost per employee)
	S2: training and qualification (KPI Ex: number of annual trainings per employee)
	S3: maturity of the workforce (KPI Ex: percentage of the workforce over 60 years old)
	S4: staff turnover (KPI Ex: percentage of employees leaving per total full-time employees)
	S5: percentage of certified sites corporates (e.g., based on ISO 14001, ISO 26000 [89,90], etc.)
	S6: total spending on product safety/revenue
	S7: total cost of relocation (e.g., hiring, training, consulting)
	S8: total R&D expenses as a percentage of total revenues
	S9: percentage of satisfied customers as of total customers/patients
	S10: percentage of revenues from repeat business as of total business
**Governance KPIs**	G1: litigation risks (KPI Ex: number of lawsuits related to anti-competitive behavior)
	G2: corruption (e.g., percentage of revenues in regions with corruption)
	G3: contributions to political parties as percentage of revenues
	G4: total number of executives (male and female employees)
	G5: staff analysis employees (per age)
	G6: staff analysis policy maturity
	G7: employees trainings in all policies and procedures regarding business ethics and anti-corruption
	G8: annual number of whistleblower schemes
**Digital Transformation KPIs**	DT1: return on digital investments
	DT2: revenue from new digital channels
	DT3: adoption and performance metrics
	DT4: patients’ experience metrics
	DT5: percentage of AI-enabled businesses
	DT6: reliability and availability
	DT7: cost–benefit analysis
	DT8: revenue from digital technology
	DT9: percentage of cloud deployments
	DT10: digital skills training
	DT11: digital marketing expenditure
	DT12: actions performed to digital illiteracy of patients

Source: Derived from our research.

## Data Availability

Data are contained within the article.
